# Exploration of Oxygen-Induced Retinopathy Model to Discover New Therapeutic Drug Targets in Retinopathies

**DOI:** 10.3389/fphar.2020.00873

**Published:** 2020-06-11

**Authors:** Maria Vähätupa, Tero A. H. Järvinen, Hannele Uusitalo-Järvinen

**Affiliations:** ^1^Faculty of Medicine and Health Technology, Tampere University, Tampere, Finland; ^2^Department of Orthopedics and Traumatology, Tampere University Hospital, Tampere, Finland; ^3^Eye Centre, Tampere University Hospital, Tampere, Finland

**Keywords:** hypoxia, angiogenesis, vascular permeability, R-Ras, filamin, myosin, retinopathy of prematurity (ROP), diabetic retinopathy

## Abstract

Oxygen-induced retinopathy (OIR) is a pure hypoxia-driven angiogenesis model and the most widely used model for ischemic retinopathies, such as retinopathy of prematurity (ROP), proliferative diabetic retinopathy (PDR), and retinal vein occlusion (RVO). OIR model has been used to test new potential anti-angiogenic factors for human diseases. We have recently performed the most comprehensive characterization of OIR by a relatively novel mass spectrometry (MS) technique, sequential window acquisition of all theoretical fragment ion mass spectra (SWATH-MS) proteomics and used genetically modified mice strains to identify novel molecular drug targets in angiogenic retinal diseases. We have confirmed the relevance of the identified molecular targets to human diseases by determining their expression pattern in neovascular membranes obtained from PDR and RVO patients. Based on our results, crystallins were the most prominent proteins induced by early hypoxic environment during the OIR, while actomyosin complex and Filamin A-R-Ras axis, that regulates vascular permeability of the angiogenic blood vessels, stood out at the peak of angiogenesis. Our results have revealed potential new therapeutic targets to address hypoxia-induced pathological angiogenesis and the associated vascular permeability in number of retinal diseases.

## Introduction

**Figure 1:**
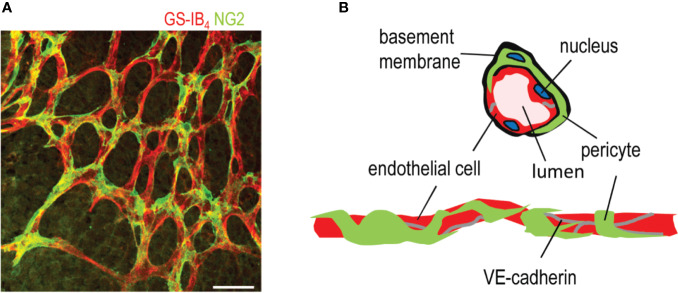
Structure of a capillary blood vessel in retina. **(A)** Capillary plexus of the mouse retina, where ECs are stained in red (Isolectin B_4_), and pericytes are stained in green (NG2 antibody). Scale bar is 50 µm. **(B)** Structure of capillary blood vessels in retina. This figure is reproduced from Vähätupa, 2019 with the permission of the copyright holder.

The formation of new blood vessels, angiogenesis, is essential for normal development, and functional blood vessels are needed for the maintenance of tissue homeostasis (**Figure 1**). Angiogenesis is controlled by a delicate balance of pro- and anti-angiogenic factors in human body. The formation of pathological neovascularization (NV) can be induced when the balance between pro- and antiangiogenic factors shifts. NV is a common factor in several retinal diseases, such as retinopathy of prematurity (ROP), diabetic retinopathy (DR), and the wet form of age-related macular degeneration (AMD) (Campochiaro, 2013). These retinal diseases are major causes of severe visual impairment and blindness in developed countries. Due to aging population, incidence of retinal diseases involving ocular NV is constantly increasing.

NV can arise either from retina or choroid in the eye. Retinal NV is seen in ischemic retinopathies, such as ROP, DR, and retinal vein occlusion (RVO), whereas choroidal NV develops in wet AMD, where choroidal neovessels grow through Bruch's membrane toward subretinal space and outer retina (Campochiaro, 2015). The neovessels are unstable and hyperpermeable, and accordingly, they leak causing hemorrhages and edema. Ultimately in wet AMD subretinal and intraretinal leakage from the neovessels leads to scarring and permanent photoreceptor damage in the retina resulting in vision loss. In DR, ROP, and RVO, retinal neovascularization is associated with hemorrhages, fibrovascular proliferation, and subsequent contraction of neovascular membranes ultimately leading to retinal detachment and blindness.

Vascular endothelial growth factor-A (VEGF-A) is a major proangiogenic factor driving angiogenesis. When the cells experience hypoxia, it stabilizes hypoxia inducible factor-1α (HIF-1α) (Lee et al., 2019; Yeo, 2019; Liao and Zhang, 2020). Stabilized HIF-1α translocates to the cell nucleus and forms a complex with HIF-1β, creating HIF-1 transcription factor. HIF-1 transcription factor then binds to hypoxia response element in the genes that promote survival in low-oxygen conditions. Active HIF-1 signaling triggers the production of large number of angiogenic growth factors, among them VEGF. These soluble growth factors direct the sprouting of the new blood vessel, i.e., angiogenesis, to deliver oxygen and address the hypoxia in the tissue (Lee et al., 2019; Yeo, 2019; Liao and Zhang, 2020). VEGF also induces vascular permeability and leakage in ocular NV diseases (Ferrara et al., 2003; Penn et al., 2008). The use of VEGF inhibitors as an antiangiogenic treatment for ocular neovascular diseases has revolutionized the treatment of these diseases and improved their prognosis dramatically. Despite the progress, there are nonresponsive patients as well as many unwanted side-effects (Yang et al., 2016). Furthermore, eradication of the neovessels by VEGF inhibitors may worsen the underlying ischemia and drive the formation of new, leaky blood vessels by alternative molecular mechanism. On the other hand, we have learned to understand that the persistency of angiogenic blood vessels leads to the progression of NV retinal diseases instead of resolving them (Mishra, 2016). Thus, the proposed molecular mechanism for future antiangiogenic therapies is one where the angiogenic blood vessels are “normalized” to stable ones to alleviate the hypoxia and stop the detrimental aberrant vascular leakage which leads scarring in retinal NV diseases (Goel et al., 2012). Thus, more effective and specific therapies that address the permeability are needed for neovascular diseases.

**Figure 2:**
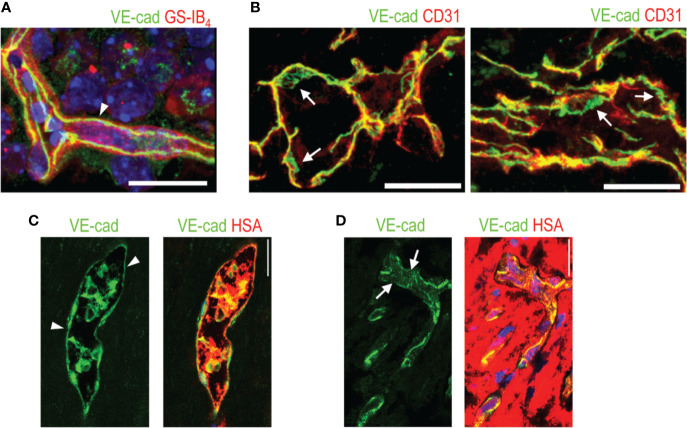
VE-cadherin expression in normal retina and in human PDR**. (A)** VE-cadherin (arrowhead, green) exhibits uniform expression in healthy mouse retina ECs (red). **(B)** VE-cadherin expression is aberrant in the vasculature of human PDR samples, (arrow). **(C)** In blood vessels where normal or continuous (arrowhead) VE-cadherin expression is seen, human serum albumin (HSA, red) is seen inside the blood vessels. **(D)** In the areas of discontinuous or absent VE-cadherin (arrows), HSA is seen around the blood vessels, indicating leakage. Scale bar is 200 µm. This figure is reproduced from Vähätupa, 2019 with the permission of the copyright holder.

Vascular permeability is strictly controlled during physiological conditions, but this control is lost in many diseases and the blood vessels become hyperpermeable. VEGF-A is the most potent growth factor in inducing vascular permeability. As a matter of fact, it was first discovered as a vascular permeability factor, a soluble protein secreted by tumors and shown to significantly increase vascular permeability (Senger et al., 1983). It is still considered as the major growth factor regulating vascular permeability in tumors and NV diseases. Features of hyperpermeable blood vessels, such as damage to the glycocalyx lining the inner lumen of endothelial cells, disorganization of cell-cell junctions and the dropout of pericytes and discontinuous endothelial cells, are well-established (Sawada et al., 2012, Butler et al., 2020). VE-cadherin is a dimeric transmembrane protein that clusters at cell-cell contacts, where it forms complexes with other signaling proteins, such as β-catenin, p120, and plakoglobin (Dejana et al., 2008) (**Figure 2**). VE-cadherin is needed for the maintenance of a stable vascular system. It controls endothelial vascular permeability and prevents excess vascular growth (Giannotta et al., 2013) (**Figure 2**).

### Oxygen-Induced Retinopathy (OIR)

**Figure 3:**
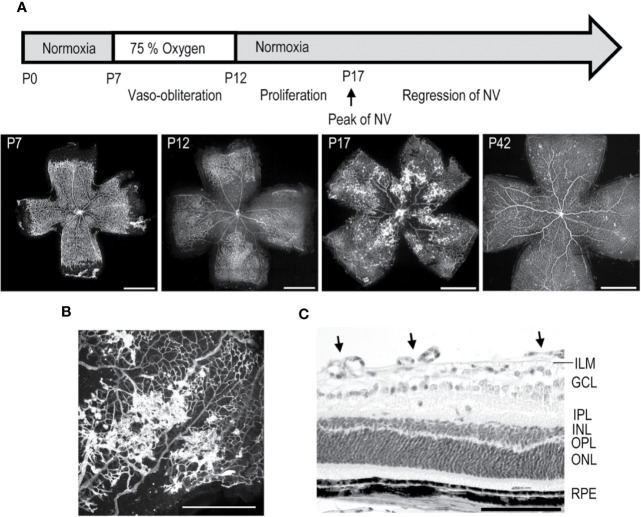
Mouse oxygen-induced retinopathy model. **(A)** Timeline of the OIR model. Induction; mice are exposed to 75% oxygen from P7 to P12 in hyperoxia chamber and returned to normal room air. Avascular area in the central retina (at P12) induces revascularization, and peak of preretinal NV is seen at P17. **(B)** Preretinal neovascular tufts form at the border between the vascular and avascular retina. **(C)** Retinal cross-section of OIR retina at P17, where preretinal tufts are sprouting towards the vitreous. Moreover, thinning of INL and OPL layers is seen. Scale bars are 1 mm in A, 500 µm in B, and 100 µm in C. This figure is reproduced from Vähätupa, 2019 with the permission of the copyright holder.

In order to find out completely novel and specific drug targets for retinal NV diseases, we have explored the most common *in vivo* model for retinal NV, the mouse oxygen-induced retinopathy (OIR) model (Smith et al., 1994) (**Figure 3**). The OIR model is widely used to study retinal NV diseases, because it shares many hallmarks with human ischemic retinopathies (Scott and Fruttiger, 2010; Stahl et al., 2010; Vessey et al., 2011; Vähätupa et al., 2016; Liu et al., 2017; Sun and Smith, 2018). The practicability of mouse OIR model has been demonstrated as it was widely used to test new potential antiangiogenic factors for human diseases and proved to provide similar outcome of the treatment as was later obtained in humans. Furthermore, it has proved feasible to test the effect of specific genes in the pathogenesis in retinal NV diseases as genetically modified mouse strains (knockout or transgenic) can be tested in it. We have performed the most comprehensive proteomics characterization of the OIR to date in order to understand molecular processes that drive the pathological neovessel formation in the model and correlated these finding with samples from human NV retinal diseases (Vähätupa et al., 2018a).

The mouse OIR model, takes advantage of plasticity of the neonatal mouse retinal blood vessels which undergo regression when the mouse is exposed to hyperoxic stimulus (Benjamin et al., 1998; Vähätupa et al., 2020). In the OIR model, neonatal mice are placed to 75% hyperoxic chamber at postnatal day 7 (P7) for five days, after which they are returned to normal room air (Smith et al., 1994; Vähätupa et al., 2016; Vähätupa et al., 2020) (**Figure 3**). Upon return to normoxic conditions, the avascular retina becomes hypoxic triggering revascularization of the retina from the periphery towards the central retina. Due to excessive hypoxic stimuli, some of the retinal blood vessels start to sprout towards the vitreous, forming preretinal NV, called preretinal tufts, that are immature and hyperpermeable (**Figure 3**). Using the OIR model, both the rate of revascularization and the amount of pathological NV can be measured (Connor et al., 2009; Stahl et al., 2010; Vähätupa et al., 2020) (**Figure 3**).

To understand the complex molecular events that drive pathological angiogenesis in the OIR model and to hopefully identify novel therapeutic target molecules for human NV retinal diseases, we investigated the pathogenesis of the mouse OIR model using the SWATH-MS full proteome-based approach (Vähätupa et al., 2018a). Overall, we were able to quantify almost 3,000 unique proteins and their expression levels during the OIR pathogenesis. Quite strikingly, the proteomics analysis revealed that the strongest cause for the differences in the protein expression levels appears to be the developmental stage of the retina. On the other hand, the pathway analysis identified angiogenesis as a mechanism that induced the changes in the protein expression levels at P17, i.e., the peak of angiogenesis in OIR (Vähätupa et al., 2018a).

### Hypoxia-Induced Expression of Crystallins in OIR

To gain more insight to the role of hypoxia in human retinopathies, we explored protein expression profile in OIR 24 h after return to normoxia. We detected a family of proteins, crystallins that were greatly upregulated (based on fold-change) by sudden hypoxia placed on retina (Vähätupa et al., 2018a). Remarkably, the crystallin family clearly stands out from the rest of the proteome by the strong hypoxia-induced expression (Vähätupa et al., 2018a). Crystallins are small heat-shock proteins that play roles in neuroprotection, because they protect cells from hypoxia and maintain mitochondrial homeostasis (Diokmetzidou et al., 2016; Kannan et al., 2016). Crystallins are expressed in the retina, where they participate in various biological processes, among them the development of retinal vasculature (Zhang et al., 2005; Sinha et al., 2008). α-crystallins may have therapeutic value in the retina. The administration of α-crystallins inhibited retinal degeneration, protected retinal ganglion cells from apoptosis, and promoted axonal regeneration in experimental animal models (Nagaraj et al., 2016). It is thought that αA-crystallin might be useful in the treatment of early DR, because adenovirus-mediated delivery of αA-crystallin inhibited pericyte dropout and vascular leakage from angiogenic blood vessels (Kim et al., 2012).

In addition to their roles in neuroprotection and development, some of the crystallins have been reported to play a prominent role in pathology of retinal NV diseases. Namely, αB-crystallin is a modulator of angiogenesis because it functions as a chaperone for VEGF and is needed for its adequate folding and secretion (Kase et al., 2010; Kannan et al., 2012). Accordingly, αB-crystallin is expressed in pathological blood vessels in PDR and is also found in vitreous fluid, and its levels correlate with VEGF levels (Chen et al., 2017). Mice deficient for αB-crystallin had less VEGF and NV compared to WT mice in the OIR model. αB-crystallin has also been reported to participate in the epithelial-mesenchymal transition (EMT), leading to TGF-β driven subretinal fibrosis in experimental AMD (Ishikawa et al., 2016). The expression of crystallins is greatly increased in many ocular diseases, such as AMD, DR, mechanical trauma, and ischemic insults (Kase et al., 2011; Thanos et al., 2014; Kannan et al., 2016). To make the matters even more complicated, an experimental diabetes study reported overexpression of α, β, and γ-crystallins in the diabetic retina, but they had lost their neuroprotective functions due to diabetes (Losiewicz and Fort, 2011).

In view of the animal studies and clinical data as well as the opposite functions of different individual crystallins and the fact that the expression levels of almost all crystallin family members are increased by the hypoxia in OIR, their potential therapeutic value is complicated (Vähätupa et al., 2018a). It seems that the therapeutic potential of crystallins needs to be investigated individually to identify the potential therapeutic crystallins among the family. Furthermore, their opposing biological functions indicate that while some members might have therapeutic value, some might have to be inhibited to treat the NV retinal diseases.

### Leakage of Plasma Proteins From Pathological Blood Vessels in OIR—Important Players in Pathobiology?

Pathological vascular permeability is a hallmark of the angiogenic blood vessels also in human retinopathies. Among the most upregulated proteins at the peak of angiogenesis in OIR (at P17) were plasma proteins such as Vitamin-D-binding protein (Gc), Albumin, and Apolipoprotein A1 (Apoa1) (Vähätupa et al., 2018a). This is most likely due to increased plasma leakage from hyperpermeable, angiogenic blood vessels. However, these proteins might also have biological functions in the OIR pathogenesis. The most upregulated protein based on fold change at P17 was Gc, a vitamin D-binding protein. It was substantially more upregulated than serum albumin, the most abundant blood protein in mammals, which may indicate selective accumulation (Vähätupa et al., 2018a). Gc is a multifunctional glycoprotein and a member of the albumin superfamily of binding proteins. It is an important carrier of vitamin D metabolites, such as calcitriol, the active form of vitamin D (Delanghe et al., 2015; Duchow et al., 2019). Interestingly, calcitriol has been reported to be antiangiogenic in a dose dependent manner in the OIR model (Albert et al., 2007). Other vitamin D receptor agonists also attenuated ocular angiogenesis in a zebrafish larvae model (Merrigan and Kennedy, 2017). Gc can also modulate inflammatory functions since it can be converted to a vitamin-D-binding protein-macrophage activating factor (DBP-MAF), which may have antitumorigenic and antiangiogenic effects (Kanda et al., 2002; Kisker et al., 2003). The antiangiogenic functions of DBP-MAF are based on its ability to inhibit VEGF signaling by decreasing the phosphorylation of VEGFR2 and ERK1/2 (Kalkunte et al., 2005). Vitamin D metabolism has been linked to ocular disease pathology and low serum levels of vitamin D represent a risk factor for AMD, DR, and glaucoma (Layana et al., 2017; Skowron et al., 2018). Vitamin D receptor (VDR) is expressed in neurons, retinal, and choroidal ECs, and especially strongly in pericytes (Jamali et al., 2017; Layana et al., 2017). Mice deficient for VDR had an increased number of pericytes and impaired NV and were resistant to the antiangiogenic functions of calcitriol in OIR (Jamali et al., 2017). It has also been suggested that vitamin D could work by protecting against oxidation and inflammation in NV retinal diseases (Layana et al., 2017; Skowron et al., 2018).

Based on the results obtained, it is possible that the upregulation of Gc in OIR at P17 could be an endogenous signal to suppress angiogenesis or a response to low levels of vitamin D metabolites in the retina. A proteomic study of ROP showed that Gc was one of the proteins found in the vitreous of ROP patients but not in the healthy controls (Sugioka et al., 2017). On the other hand, the increased amount of Gc in NV Retina could also be outcome of the increased expression, not just the product of enhanced leakage, because ECs release Gc under stress, but not during normal growth (Raymond et al., 2005). Gc works as a growth factor in blood vessels and induces cell migration and proliferation of vascular SMCs at the site of vascular injury (Raymond et al., 2005). Recently published meta-analysis indicated that Gc polymorphisms play important roles in cancer pathogenesis (Zhu et al., 2019). The role of Gc in ocular angiogenesis and OIR has not been investigated. Thus, further studies are needed to find out whether its enhanced accumulation is the by-product of leakage or whether it is produced by the ECs in angiogenic blood vessels. These issues would clarify its role in neovascular ocular diseases.

### Proteins Involved in Mechanotransduction Are Upregulated in OIR and Angiogenesis in OIR May Involve Endothelial-to-Mesenchymal Transition (EndMT)

When we focused on the peak of angiogenesis in OIR, i.e., P17, majority of the upregulated proteins were involved in the mechanotransduction, cell adhesion, and actin cytoskeleton signaling (Vähätupa et al., 2018a). Proteins associated to actin cytoskeleton play an important role in cell-cell adhesion (Hoelzle and Svitkina, 2012) and actin cytoskeleton remodeling is implicated as an important factor in angiogenesis (Keezer et al., 2003; Kilarski et al., 2003). In order to change shape, cells need to sense and respond to external mechanical stimulus and forces. These molecules are thought to be part of the mechanobiome, which includes molecules ranging from cell adhesion to ECM to the contractile molecules in the cytosol (Kothari et al., 2019). Together they form a network of proteins, where some act either as a sensors or actuators. Some of the proteins, like non-muscle myosin II, can act both as a sensor and an actuator, and further link to other proteins like scaffolding proteins and transcription factors (Luo et al., 2012; Kothari et al., 2019). Notably, the actomyosin network is a significant driver of cellular processes by regulating gene expression and has been implicated as an important regulator in angiogenesis (Angulo-Urarte et al., 2018; Kothari et al., 2019).

Actomyosin (complex of myosin and actin) contraction-based pulling forces at the cell junctions are regulating the switch between stable and unstable EC junctions (Angulo-Urarte et al., 2018).

One of the molecules involved in this process is Vinculin, a focal adhesion protein connecting cell adhesions to the actin cytoskeleton (Ziegler et al., 2008). Vinculin associates with VE-cadherin on the EC junctions as a regulator force-dependent remodeling (Huveneers et al., 2012) and deregulation of Vinculin is associated with enhanced cancer cell migration (Goldmann et al., 2013). Interestingly, Vinculin was one of the proteins upregulated at the angiogenic phase in OIR (Vähätupa et al., 2018a).

**Figure 4:**
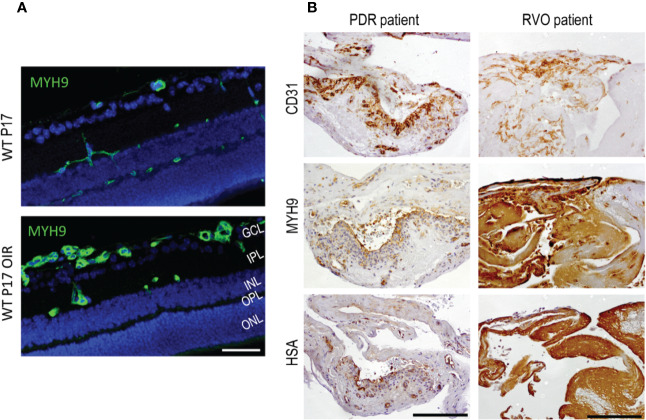
Myosin IIA (Myh9) is expressed in OIR retinas and human PDR and RVO fibrovascular membranes. **(A)** Mouse retinal sections stained for Myh9 (green) exhibit increased expression in the blood vessels in OIR retinas. **(B)** The blood vessels (CD31) had HSA restricted inside the blood vessels when Myh9 expression was low. Strong Myh9 expression is associated with strong HSA accumulation throughout the sample in fibrotic RVO sample. In PDR samples with low Myh9 expression in the blood vessels (CD31) had HSA restricted inside the blood vessels. In fibrotic RVO sample strong Myh9 expression together with strong HSA accumulation throughout the sample was seen. Scale bar is 50 µm in A and 200 µm in B. This figure is reproduced from Vähätupa, 2019 with the permission of the copyright holders.

Among the mechanobiome proteins upregulated in OIR (Vähätupa et al., 2018a), Myh9 is especially relevant (**Figure 4**). We demonstrated strong induction of Myh9 in OIR at the peak of angiogenesis, i.e., at P17 (Vähätupa et al., 2018a). Strikingly, the strong Myh9 expression took place selectively in angiogenic blood vessels in the OIR (Vähätupa et al., 2018a) (**Figure 4**). Myh9 encodes for a myosin IIA heavy chain, a cytoskeletal contractile/motor protein, which participates in a variety of processes requiring contractile force, such as cytokinesis, cell migration, polarization and adhesion, and the maintenance of cell shape (Pecci et al., 2018). Its expression levels are considered to reflect the stiffness of the tissue, such that the stiffest tissues express high levels of Myh9 (Irianto et al., 2016). When cells are migrating, they adhere to the extracellular matrix, which transmits forces inside the cell, and the nonmuscle myosins balance the mechanical forces. The stiffer the tissue, the more contractility the migrating cells need. ECs form contact with each other with lamellipodia like structures. Then they retract and remain in contact with thin bridges formed by filopodia-like protrusions, which connect to neighboring ECs by VE-cadherin-rich junctions. Actin bundles in bridges recruit nonmuscle myosin II and mature into stress fibers. Myosin II activity is important for bridge formation and accumulation of VE-cadherin in nascent adherens junctions (Hoelzle and Svitkina, 2012). When the ECs are migrating, these myosin IIA-generated forces destabilize endothelial cell-cell junctions and increase vascular permeability (Huynh et al., 2011; Bordeleau et al., 2017; Lampi and Reinhart-King, 2018). Myh9 is overexpressed in many cancers, and its high expression is associated with poor prognosis indicating very aggressive phenotype of the tumor (Newell-Litwa et al., 2015). The complex interactions between the stiffness of the tissue and myosin IIA was recently elegantly demonstrated in glioblastoma (Picariello et al., 2019; Surcel and Robinson, 2019). The deletion of Myh9 led to the inhibition of tumor cell invasion, but its effects on cell proliferation are different depending on environmental mechanics, i.e., whether the cells are on soft or stiff surface (Picariello et al., 2019). In addition to regulating the endothelial cell-cell junctions, Myh9 regulates cell migration in angiogenesis (Vicente-Manzanares et al., 2009; Yoon et al., 2019). Myh9 is required for sprouting angiogenesis as it is crucial for mediating mechanical forces and maintaining cell–cell contacts between tip and stalk cells (Yoon et al., 2019). This is the likeliest explanation for the induction Myh9 during sprouting angiogenesis in OIR (Vähätupa et al., 2018a) (**Figure 4**).

As the mechanobiome is an important regulator of gene expression, the activation of Myh9 led to elevated VEGF expression and induction of arteriogenesis in the ischemia-driven arteriogenesis (Morrison et al., 2014). Myh9 has also been shown to control the translocalization of nucleolin, which is primarily a nuclear protein but translocates to the EC surface in angiogenesis (Christian et al., 2003; Huang et al., 2006; Fogal et al., 2009; Li et al., 2018). Therapies that antagonize nucleolin or block Myh9 inhibit angiogenesis by causing EC apoptosis and normalizing the vasculature (Fogal et al., 2009). In our proteomic study, we observed only a modest increase in the nucleolin expression (Vähätupa et al., 2018a). However, the gene expression levels are not relevant to function of nucleolin in angiogenesis, because its function is related to its translocation to cell surface. It was recently shown that nucleolin is translocated to the EC surface in neovascular blood vessels in OIR, whereas it stayed nuclear in the normoxic conditions (Garfias et al., 2019). Similar nucleolin translocation takes place in corneal neovascularization and nucleolin-binding DNA aptamer was able to inhibit corneal neovascularization (Vivanco-Rojas et al., 2020).

We also studied Myh9 expression in human NV retinal diseases. Strong Myh9 expression correlated with HSA accumulation (i.e., leakage) in PDR and RVO membranes (Vähätupa et al., 2018a) (**Figure 4**). This indicates increased EC contractility, which in turn, results in increased vascular permeability. Myh9 is also expressed in fibroblast like cells that have undergone endothelial to mesenchymal transition (EndMT) (Evrard et al., 2016). Actin cytoskeleton remodeling is well characterized process in epithelial to mesenchymal transition in cancer invasion and metastasis (Peng et al., 2018).

In EndMT, cells start to express mesenchymal markers, gain increased motility, and begin the secretion of extracellular matrix proteins. This phenomenon is reported to contribute to endothelial dysfunction during inflammation (Cho et al., 2018) and it is involved in a variety of disease processes, such as vascular or tissue fibrosis, and in the tumor environment (Hong et al., 2018). Upstream regulator analysis of OIR proteomics data indicates TGF-β, Mkl2, and Mknk1 as potential enhancers of increased angiogenesis in OIR. In addition, TGF-β is known for its roles in the induction of EMT and myofibroblast transformation. Mkl2 (and Mkl1) are key regulators in these cellular processes (Crider et al., 2011; Gasparics and Sebe, 2018). Increased TGF-β signaling leads to EndMT in the vasculature, which shares many features with EMT (Dejana et al., 2017; Schwartz et al., 2018). Enhanced vascular TGF-β signaling may also induce fibrosis to surrounding tissue. EndMT takes place in glucose-treated retinal ECs, where EC damage leads to a decrease in endothelial markers and an increase in mesenchymal markers, processes which is induced by TGF-β (Cao et al., 2014; Thomas et al., 2019). In light of the recent finding that TGF-β together with αb-crystallin is associated with EMT and with subretinal scarring (Ishikawa et al., 2016), simultaneous up-regulation of both molecules could provide hints about the potential events that ultimately lead to fibrosis in angiogenic retinal diseases. Furthermore, it was recently demonstrated that myeloid cell-derived furin, TGF-β activator, is crucial for the angiogenesis in OIR (Vähätupa et al., 2018b), but furin also aggravates hypoxia-induced blood-brain barrier dysfunction (Baumann et al., 2019). Taking together, the upregulation of proteins involved in mechanotransduction and actomyosin network as well as in the TGF-β signaling in OIR during the angiogenic phase may suggest that these processes could be important for angiogenesis, and thus provide a potential therapeutic target.

### R-Ras and Filamin A Axis in Vascular Stabilization of Angiogenic Blood Vessels in OIR

One of the proteins predicted to be causing enhanced angiogenesis in the OIR model was Flna, which is the most abundant member of the filamins. Filamins act as actin binding scaffold proteins that maintain ECM connections. They act as mechanoresponsive actin crosslinkers and are important players in the actomyosin network and crucial for mechanotransduction (Baudier et al., 2018; Balassy et al., 2019). Thus, they modulate the interactions between actin cytoskeleton and the ECM, enabling changes in the transmission of forces at the cell periphery (Razinia et al., 2012).

**Figure 5:**
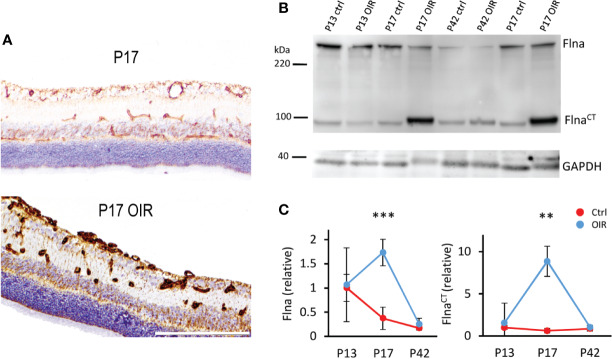
Filamin A upregulation in OIR retinal blood vessels. **(A)** Retinal sections stained for Flna (brown) show induction of Flna in retinal blood vessels in OIR. **(B)** Western blot of control and OIR retinas at different time points shows increase of cleaved Flna fragment, Flna_CT_ in OIR at P17. **(C)** Quantification of total Flna and Flna_CT_ in control and OIR retinas. Scale bar is 200 µm. This figure is adapted from Vähätupa, 2019 with the permission of the copyright holders **P < 0.01, ***P < 0.001.

We showed that Flna was expressed in the retinal blood vessels and the expression was increased in OIR (**Figure 5**). In addition to the increase in the levels of total Flna, a 14-fold induction of C-terminal Flna fragment (Flna^CT^) was detected (Vähätupa et al., 2018a) (**Figure 5**). In hypoxia, Flna is rapidly upregulated and then interacts with HIF-1α and promotes angiogenesis (Zheng et al., 2014). In hypoxia, Flna undergoes calpain dependent cleavage, and its 90 kDa C-terminal fragment (Flna^CT^) accumulates into the nucleus, enhancing HIF-1α (but not HIF-2α) accumulation there. Flna^CT^ interaction with HIF-1α localizes the HIF-1 complex to the promoter regions of HIF-1 target genes and enhances their transcription/expression (Zheng et al., 2014). Upregulation of Flna has been found in many cancers, and tumor cells lacking Flna exhibits impaired growth and angiogenesis, as well as the reduced expression of HIF target genes (Nallapalli et al., 2012; Zheng et al., 2014). Targeting Flna has been proposed as a target for cancer therapy (Nallapalli et al., 2012). Considering that N-terminal Flna is needed for the stability of the EC barrier, specific inhibition of Flna^CT^ could provide a more potent antiangiogenic therapeutic effect than general Flna inhibition. Indeed, it was recently shown that blocking the calpain-dependent cleavage of Flna hinders the growth of tumor cells (Salimi et al., 2018).

**Figure 6:**
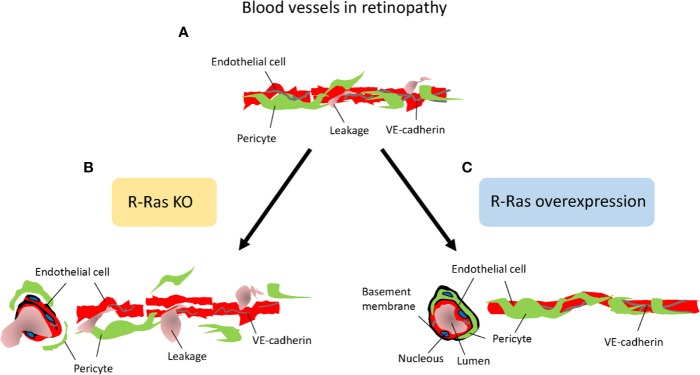
R-Ras is the master regulator of vascular permeability. Schematic presentation on the role of R-Ras in regulating vascular permeability. **(A)** Angiogenic blood vessels in retinopathy are hyperpermeable and leak. **(B)** R-Ras deficiency leads to reduced pericyte and VE-cadherin coverage on endothelial cells, and thus enhanced plasma leakage. **(C)** Increasing R-Ras expression normalizes pathological blood vessels, leakage is reduced and proper perfusion established in the blood vessels.

Concerning molecular mechanism by which Flna induces its effects on blood vessels, it interacts with small GTPase R-Ras to control cell migration and maintain vascular barrier function (Griffiths et al., 2011). R-Ras is a member of the Ras superfamily of known oncogenes. However, R-Ras maintains cellular quiescence and inhibits cell proliferation, effects that are opposite to the biological functions of other Ras family members, which play a prominent role in cancer progression (Prior et al., 2012; May et al., 2015). Generation of R-Ras deficient mice revealed that the primary function of R-Ras is in the regulation blood vessel maturation and angiogenesis (Komatsu and Ruoslahti, 2005; Sawada et al., 2012; Sawada and Komatsu, 2012) (**Figure 6**). R-Ras inhibits vessel sprouting and branching and EC migration (Sawada et al., 2012; Sawada et al., 2015b). Most importantly, it is a crucial gene to regulate vascular permeability not only during pathological tumor angiogenesis, but also in physiological tissue repair, that is, in skin wounds as it stabilizes blood vessel walls (Komatsu and Ruoslahti, 2005; Sawada et al., 2012; Sawada et al., 2015a; Ketomäki et al., 2019; Järvinen, 2020) (**Figure 6**). R-Ras is also integral for the proper lumenization of the angiogenic blood vessels, i.e., it restores proper lumen formation and normal perfusion and most importantly addresses the hypoxia (Li et al., 2017). Taken together, R-Ras is considered as a master regulator of vascular permeability in angiogenesis (**Figure 6**).

R-Ras is crucial for blood vessel integrity and stabilization in the OIR model, where R-Ras deficient mice have a hyper-permeable phenotype (Vähätupa et al., 2016) (**Figure 6**). Although the revascularization rates are identical between WT and R-Ras KO mice, the neovascular blood vessels leak twice as much in the KO mice than in the WT mice. The pericyte coverage is reduced in the angiogenic blood vessels in OIR in the R-Ras KO mice, and VE-cadherin expression is reduced (Vähätupa et al., 2016). R-Ras expression in human DR samples showed that the immature, pathological VEGFR2+ blood vessels lack R-Ras expression and R-Ras expression correlates inversely with leakage from the immature blood vessels, i.e., more leakage in human DR, less R-Ras is expressed in the blood vessels (Vähätupa et al., 2016). The molecular mechanisms by which R-Ras exerts its function on vascular permeability are not known in detail, but our understanding on its function is rapidly emerging as its central role in regulating vascular permeability is appreciated. R-Ras is functionally different from classic antiangiogenic agents as it does not induce endothelial cell apoptosis, but it actually promotes endothelial cell survival (Komatsu and Ruoslahti, 2005; Takino et al., 2019).

Concerning endothelial cell death, Ras guanyl nucleotide releasing protein 2 (RasGRP2) inhibits EC apoptosis *via* R-Ras (Takino et al., 2019). Furthermore, R-Ras interacts with Flna and co-localizes into plasma membrane (Griffiths et al., 2011). Disrupting this interaction promotes disorganization of VE-cadherin at adherens junctions and leads to leaky blood vessels. R-Ras binds to Flna in its N-terminus (Griffiths et al., 2011). Thus, the selective blockage of either the cleavage of Flna (that creates angiogenic Flna^CT^) or Flna^CT^ fragment itself could be a potential therapeutic approach in ischemic retinopathies involving NV, because it should not interfere with the physical interaction between R-Ras and N-terminus of Flna that maintains vascular stability in blood vessels. Interestingly, we showed that the induction of Flna^CT^ is almost 15-fold in OIR (Vähätupa et al., 2018a), making it viable molecular target to inhibit sprouting angiogenesis. Furthermore, actin-binding protein Girdin/GIV regulates transendothelial permeability by also controlling VE-cadherin trafficking through R-Ras (Ichimiya et al., 2015). The mechanism of action regulating vascular permeability could also be related to R-Ras being also an integrin activator (Zhang et al., 1996). Notch activates in R-Ras – β1 integrin pathway in radial glia (Fujita et al., 2020). Notch signaling tells endothelial cells to stop migrating and proliferating in angiogenesis (Luo et al., 2019). Notch induced activation of R-Ras – β1 integrin pathway could be the mechanism for the appearance of R-Ras during blood vessel maturation (Komatsu and Ruoslahti, 2005; Vähätupa et al., 2016). Flna, in turn, interacts with β1, β2, and β7 integrin tails (Toomer et al., 2019). R-Ras regulates integrin dependent cell migration through Flna (Gawecka et al., 2010). Sandri et al. showed integrin-activated R-Ras recruits RIN2 to focal adhesions and induces RIN2 conversion to a Rab5-docking protein (Fernandez-Borja, 2012; Sandri et al., 2012). Subsequently, the complex promotes actin polymerization and formation of new focal contacts (Fernandez-Borja, 2012; Sandri et al., 2012). Finally, the vascular stabilizing functions of R-Ras also involve pericytes, as R-Ras is needed for their recruitment to the angiogenic blood vessels (Sawada et al., 2012) (**Figure 6**). Pericyte loss, in turn, leads to pathological angiogenesis, and their loss is considered as a key feature in the progression of DR (Beltramo and Porta, 2013; Mishra, 2016).

Our understanding on role of R-Ras as a master regulator of vascular permeability is currently increasing in diseased states. It was recently demonstrated that R-Ras induced different phosphoinositide 3-kinase-(PI3K)-Akt signaling than VEGF-A and these two different PI3K-Akt signaling “phenotypes” lead to totally different phenotypes in angiogenic blood vessels in hypoxia (Li et al., 2017). Whereas VEGF-A induced PI3K-Akt signaling leads to sprouting angiogenesis, R-Ras induced signaling leads to upregulation of podocalyxin and proper lumenogenesis of the angiogenic blood vessels (Li et al., 2017). Podocalyxin, in turn, is required for maintaining vascular permeability in blood-brain barrier (Cait et al., 2019), which could be the mechanism by which R-Ras exerts its effects in OIR and retinopathy. Interestingly, increased R-Ras palmitoylation is the mechanism by which R-Ras can be inactivated in vascular diseases (Wei et al., 2020). R-Ras is the molecular target of epalmitoylation enzyme acyl-protein thioesterase 1 (APT1). APT1 deficiency leads to enhanced palmitoylation of R-Ras and inhibits lumen formation, whereas the inhibition of R-Ras palmitoylation rescues vessel lumen formation (Wei et al., 2020).

The expression of another filamin-family member, Filamin B (Flnb) was increased at P17 OIR. Whereas Flna is more ubiquitously expressed in vascular mural cells and ECs, the expression of Flnb is restricted to ECs (Del Valle-Perez et al., 2010). Disruption of Flnb, but not Flna, leads to inhibition of EC migration after VEGF induction (Del Valle-Perez et al., 2010). Flnb deficiency led to an impaired microvascular network in the central nervous system (Zhou et al., 2007), whereas gain of function of Flnb is proposed to modulate the interaction between Flna and Flnb and impair filamins function in the regulation of angiogenesis, which could lead to abnormal angiogenesis. VEGF and protein kinase C promote the ubiquitination of Flnb, which leads to angiogenic-promoting HDAC7 phosphorylation (Su et al., 2013). Taking together, these results suggest, that the increased expression of Flna may play a role in the regulation of vascular permeability *via* N-terminal Flna, whereas Flna^CT^ and Flnb may drive angiogenesis and the migration of ECs in OIR. Similar to our results in OIR (Vähätupa et al., 2018a), the enhanced expression of Flna has been reported in proteomics-based studies from other models of retinal diseases (Murugesan et al., 2019; Christakopoulos et al., 2019).

## Conclusion

OIR is a hypoxia-induced angiogenesis model and a representative experimental model for several human neovascular retinal diseases. As it is acknowledged that the angiogenic blood vessels actually support the persistency of hypoxia in the tissue and lead to the progression of NV diseases instead of resolving the pathology (Mishra, 2016), it has become absolute necessity to understand their biology in detail. The most comprehensive proteomics-based analysis of the OIR to date delineates the possible molecular mechanisms that may drive angiogenesis in this model (Vähätupa et al., 2018a). Together with studies using genetically modified mice strain we were able to identify not just proteins induced in the hypoxic environment of retina, but also molecular interplay between the proteins induced by hypoxia and then by subsequent angiogenesis as well as the R-Ras – Filamin A axis that regulates vascular permeability in OIR. Interestingly, the sprouting angiogenesis in OIR needs an EC specific induction of genes involved in the mechanotransduction of forces generated by migrating cells. This network may present completely novel drug targets for future therapies. We hope that the better understanding of the molecules involved in the OIR will lead to new drug therapies for the human NV retinal diseases.

## Author Contributions

MV made the figures. All authors reviewed the literature, wrote and edited the manuscript.

## Conflict of Interest

The authors declare that the research was conducted in the absence of any commercial or financial relationships that could be construed as a potential conflict of interest.
